# The effect of vaginal delivery and Caesarean section on the anal Sphincter complex of Primipara based on optimized three-dimensional ultrasound image and nuclear regression Reconstruction Algorithm

**DOI:** 10.12669/pjms.37.6-WIT.4859

**Published:** 2021

**Authors:** Naxin He, Liang Shi

**Affiliations:** 1 Naxin He, Attending Physician. Department of Gynaecology and Obstetrics, The People’s hospital of Putuo, Zhoushan, 316100, Zhejiang, China; 2 Liang Shi, Attending Physician. Department of Gynaecology and Obstetrics, Xinchang People’s Hospital, Shaoxing, 312500, Zhejiang Province, China

**Keywords:** Algorithm, Anal sphincter complex, Caesarean section, Nuclear regression reconstruction three-dimensional ultrasound image, Vaginal delivery

## Abstract

**Objective:**

The study used the optimized nuclear regression reconstruction algorithm to explore the value of three-dimensional perineal ultrasound evaluation of the effect of caesarean delivery and caesarean section on the anal sphincter complex of primipara.

**Methods:**

This study performed three-dimensional perineal ultrasound scanning of the anal sphincter complex of 157 primiparas 42 days after delivery. Among them, 77 were in caesarean delivery (spontaneous delivery group) and 80 were in caesarean section (caesarean delivery group) from September 2018 to December 2020 in our hospital. The thickness of the end plane, the middle plane, the distal plane and the distal plane of the external anal sphincter at 3, 6, 9, 12 o’clock direction, and measure the thickness of the central plane of the pubic rectum muscle at 4, 8 o’clock direction. At the same time, the study used tomography and volume contrast imaging to observe the morphology and integrity of the anal sphincter complex.

**Results:**

The thickness of the distal anal sphincter at the 12 o’clock direction, the proximal anal sphincter at 6, 12 o’clock, and the central plane at 9 and 12 o’clock in the obstetric group were smaller than those in the caesarean section group (all P < 0.05). There were no significant differences in the thickness of the remaining anal internal and external anal sphincter and puborectalis muscles between the two groups in different directions (all P> 0.05). In the obstetric group, a perineal sphincter defect was found via three-dimensional perineal ultrasound.

**Conclusion:**

The delivery method has a certain influence on the shape of the anal sphincter complex. The thickness of the internal and external anal sphincter of the primiparous women in a certain direction is significantly smaller than that of caesarean section. Transperineally three-dimensional ultrasound can clearly show the morphological characteristics and integrity of the anal sphincter complex, and diagnose the defect of the anal sphincter complex.

## INTRODUCTION

The kernel regression algorithm includes two algorithms: traditional kernel regression (CKR) and controlled kernel regression (SKR)^1^-^3^, used for medical image processing and image reconstruction, demonstrating good denoising effects and edge preservation effects.^4^-^6^ However, the calculation process is complicated. During image reconstruction algorithm, the function is introduced to automatically extract the features of the image data, and the optimized internal parameters have higher accuracy and reliability.^7^,^8^ In this study, three-dimensional transperineally ultrasound was used to evaluate the effect of caesarean delivery and caesarean section on ASC.

## METHODS

Oner hundred fifty seven primiparas who were postnatally reviewed in our hospital from September 2018 to December 2020 were selected after IRB approval (Dated March 23, 2021), aged 25 to 40 years old, with an average of (29.3 ± 2.8) years; 77 of them were in labour delivery (shun delivery group) and 80 were in caesarean delivery (Caesarean section group).

###  Inclusion criteria

1. Single pregnancy, ultrasound in the second trimester confirmed that the foetus size is consistent with gestational week; 2. Underwent three-dimensional transperineally ultrasound examination 42 days after delivery; 3. No history of pelvic surgery. In the perinatal group, 36 patients received perineal side resection and one patient had forceps delivery; none of the women in the caesarean section group entered the second stage of labour. There was no statistically significant difference in maternal age, height, bodyweight at delivery and birth weight of new-borns between the two groups (all P> 0.05), Table-I.

**Table-I:** Comparison of general conditions of maternal and neonates between the caesarean section and the caesarean section.

*Group*	*Age (year old)*	*Height (cm)*	*Body weight at delivery / delivery (kg)*	

			*Maternal*	*New-born*
Natural birth group (n = 77)	29.4±3.0	162.91±3.69	68.78±4.53	3.22±0.30
Caesarean section group (n = 80)	30.1±3.2	163.08±4.02	69.51±2.85	3.21±0.28
t value	0.4	0.26	1.2	-0.23
P value	0.69	0.79	0.23	0.82

The use of GE Coulson E8Expert ultrasound diagnostic apparatus, cavity volume probe, frequency 5 ~ 9MHz. Instruct the subject to defecate and urinate before the inspection. During the examination, the subject was instructed to take the lithotomy position, hip flexion, and mild knee abduction. Place the probe on the perineum of the patient, take a cross-section, scan from the end of the anus to the anorectal angle, and observe the ASC in multiple planes. 1. The proximal plane, the IAS plane adjacent to the distal end of the anorectal angle; 2. The central plane, the PRM surrounds the IAS at the rear and shows the clearest plane; 3. The distal plane, at the same time clearly shows the plane of the IAS and EAS. Measure the thickness of IAS at 3, 6, 9, and 12 o’clock in the above three planes, and measure the thickness of EAS at 3, 6, 9, and 12 o’clock in the far plane.

### Statistical analysis

Use SPSS20.0 statistical analysis software. Independent sample t-test was used to compare the thickness of ASC in different directions and the general conditions of maternal and new-born between the caesarean section and the caesarean section group. P <0.05 was considered statistically significant. In the CPU version of the nuclear regression reconstruction algorithm, the algorithm itself is divided into two parts: data acquisition and target volume reconstruction.

**Fig.1:**
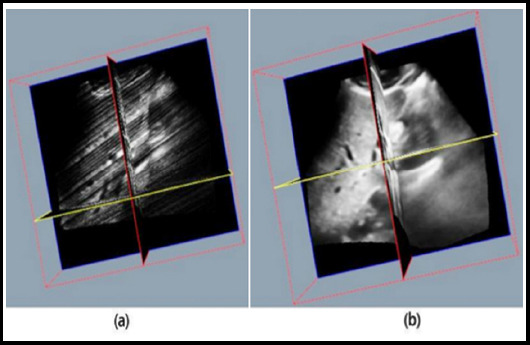
Three-dimensional figure of human organs. (a) Initial filling stage. (b) Regression stage.


After entering the initial filling stage, discrete three-dimensional ultrasound graphic data will be obtained as shown in Fig.1. The various points in this graphic can be expressed by the formula under non-parametric estimation:

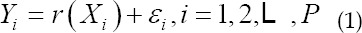


Where *Y_i_* is the observed value, *r(⋅)* is the regression equation (cannot be determined), 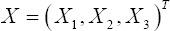 is the three-dimensional coordinate of the corresponding point, *ε_i_* is the noise error of the constant distribution that does not interfere with each other, 1 ~ P represents all points in the three-dimensional graph. It can be seen that *r(X_i_*) is assumed to be the actual value of the 3D ultrasound graph here, however, this particular function cannot be determined, Here we continue to assume that the *r(X_i_*) function is locally N order derivable. For any point X in the three-dimensional ultrasound graph, to estimate the value of the regression equation at this time, the function can be locally expanded at this point X. In general, if *(X_i_*) is a sampling point near the X point, so here can be expanded by Taylor formula for *r(X_i_*) can be expressed as:


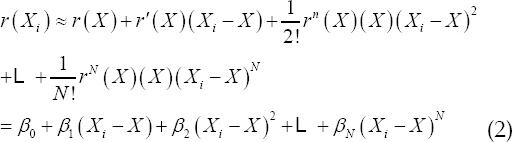


The above shows that if the Taylor function is regarded as the local performance of the regression function, the estimated parameter *β_0_* in equation (2) can be expressed as the estimated solution of the regression equation when the data is *(X_i_*).

To make the observed value *(Y_i_*) closer to the value of the regression equation *r(X_i_*), only when *ε_i_* is closer to 0, the resulting regression equation is closer to the true value. In order to solve this optimization problem, the least squares equation is used here:


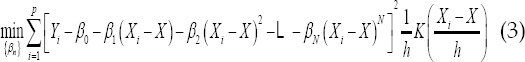


Where *K(⋅)* is a kernel function and a weight function, used to determine the influence of the points around the calculation point on the calculation point. To control this influence. In particular, function *K(⋅)* is a symmetric function, and its maximum value is 0, which satisfies:


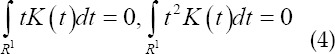


Here c is a constant value. The function *K(⋅)* selected here is usually a Gaussian function, but it can also be other forms of functions, as long as the above constraints are satisfied. In the literature on non-parametric statistics, the most widely considered are local constants, linear and quadratic approximations. The corresponding values of N are 0, 1, and 2, respectively, This paper chooses the case where N is 2.

The kernel function *K(⋅)* is now a function with three directional variables, and its bandwidth h should be a 3 * 3 matrix. However, usually the covariates of each scale have the same mean and variance. To simplify the problem, only the bandwidth h in all directions is considered here, and then the Gaussian function selected is:


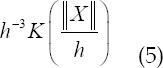


Where h is the bandwidth of the Gaussian function and the smoothing parameter introduced earlier. As a three-dimensional point X, there is 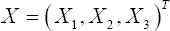. For more convenient calculation, let N take the value of 3, taking into account the situation of the three-dimensional point, the formula (3) Equivalent transformation into


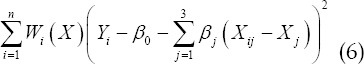


Here *W_i_(X)* represents the influence of point *X_i_* on the central point X (calculation point), and its value is determined according to the distance from point *X_i_* to point X and the corresponding bandwidth h, expressed as:


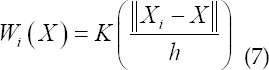


To facilitate the operation of the computer, it is necessary to convert the minimum case of equation (6) to the form of a matrix. For each point around the calculation point, there is a distance matrix *X_X_* to store the distance from the surrounding points to the calculation point. The matrix is as follows:


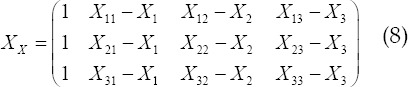


This matrix is also the distance matrix that the CPU needs to prepare for GPU operations when the GPU algorithm is implemented later. This matrix will be further introduced in subsequent articles. Suppose, when formula (6) obtains the minimum value, the corresponding value of *β_0_,β_1_,β_2_,β_3_* is:


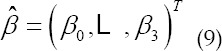


Assuming that the estimated value is 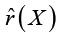, according to the 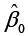 mentioned above, it is the estimated value sought in this article, it is not difficult to obtain:


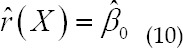


## RESULTS

**Fig.2:**
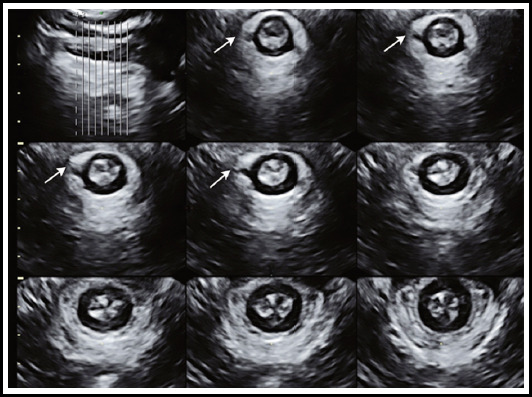
Maternity, 28 years old, 42 days after mild anal incontinence. Perineal three-dimensional ultrasound showed continuous interruption of EAS by perineum), located at 10 o’clock.

One child in the perinatal group showed a continuous interruption of EAS after three months of perineal ultrasound (Fig.2).

The results of the three groups of three-dimensional ultrasound measurement of perineal thickness of the two groups of IAS, EAS and PRM are shown in Table-II and Table-III. The thickness of the IAS proximal plane 6, 12 o’clock direction and the central plane 9 and 12 o’clock direction of the obstetric group were less than that of the caesarean section group (all P <0.05).

**Table-II:** Comparison of IAS proximal, middle and distal plane thickness measurements between caesarean section and caesarean section.

*Group*	*IAS proximal plane*			*IAS mid-plane*		

	*3 o’clock*	*6 o’clock*	*9 o’clock*	*12o’clock*	*3 o’clock*	*6 o’clock*
Natural birth group (n = 77)	2.05±0.54	1.86±0.53	2.15±0.57	1.53±0.60	2.25±0.58	1.83±0.55
Caesarean Section (n = 80)	2.21±0.67	2.11±0.76	2.17±0.58	1.78±0.55	2.40±0.59	1.73±0.48
t value	1.6	2.32	0.21	2.7	1.61	-1.19
P value	0.11	0.02	0.83	<0.01	0.11	0.23
*Group*	*IAS mid-plane*			*IAS far plane*		

	*9o’clock*	*12o’clock*	*3o’clock*	*6o’clock*	*9o’clock*	*12o’clock*
Natural birth group (n = 77)	2.33±0.51	1.65±0.63	2.13±0.60	2.04±0.59	2.23±0.51	1.55±0.56
Caesarean Section (n = 80)	2.53±0.61	2.01±0.57	2.19±0.59	1.98±0.57	2.37±0.54	1.72±0.60
t value	2.31	3.7	0.68	-0.76	1.67	1.82
P value	0.02	<0.01	0.5	0.45	0.1	0.07

**Table-III:** Comparison of thickness measurements between the distal EAS plane and the central plane of the caesarean and caesarean section.

*Group*		*EAS far plane*			*PRM Midplane*	

	*3 o’clock*	*6 o’clock*	*9 o’clok*	*12 o’clock*	*4 o’clock*	*8 o’clock*
Natural birth group (n = 77)	2.93±0.82	2.04±0.59	3.12±0.84	1.04±0.27	7.00±1.40	7.21±1.39
Caesarean Section (n = 80)	2.87±0.80	2.73±0.88	3.23±0.77	1.87±0.55	7.37±1.51	7.34±1.71
t value	-0.52	-0.48	0.8	12.15	1.62	0.55
P value	0.6	0.63	0.43	<0.01	0.11	0.58

## DISCUSSION

Obstetric anal sphincter injury occurs during delivery. Thinning of EAS will weaken the function of ASC. After delivery, IAS and EAS become thinner. Any injury of EAS, PRM, and IAS is closely related to incontinence in the anus. Meriwether et al.^9^ used transanal ultrasound and transperineal ultrasound for examinations, and the results showed that, the left PRM of normal women was thicker than the right. Johannessen et al.^10^ proposed that, changes in hormones, neuromuscular, and mechanics during pregnancy played a more important role in the basin function damage than vaginal delivery. In the study, it was found that the delivery method has a certain impact on the shape of the anal sphincter complex. In this study, the perineum of the delivery group and the caesarean section group was thicker than that of the natural delivery group at the proximal plane of IAS at 3, 6 and 9 o’clock.

The kernel regression image algorithm mainly uses the kernel regression interpolation function for non-parametric estimation. In terms of image noise reduction, image fusion, and image interpolation, this algorithm has good denoising and interpolation effects. Takeda et al.^11^ applied the kernel regression algorithm to image noise reduction. Compared with the traditional method, the image edge preservation effect and the image interpolation effect were both significant. Ultrasound is widely used in the clinic to detect the perineum. The main imaging methods for evaluating the sphincter complex are transrectal ultrasound and magnetic resonance imaging. Ultrasound imaging can present a 360-degree cross-section of anal sphincter. The anal sphincter is evaluated factoring into the proximal plane, the middle plane and the distal plane.^12^-^14^ To use kernel regression algorithm to process the ultrasound image effectively removes the blurred image and improves the diagnosis accuracy. Compared with the traditional image, the three-dimensional image in this research was clearer. Stuart et al.^15^ performed transperineal ultrasound imaging on women undergoing the repair of obstetric sphincter injury six months before. The images obtained were clear whether in contracted or resting state.

Rectal ultrasound is the gold standard for diagnosing anal sphincter injury. Some scholars believe that, the ultrasound probe will compress the anal sphincter to a certain extent and affect the measurement to a certain extent. Transperineal ultrasound is reduces the deformation of the anus, and the three-dimensional tea-red color is the basin imaging diagnosis mode. Williams et al.^16^ found that, three-dimensional ultrasound and 3D technology imaging had a high degree of conformity in the measurement of EAS, and the IAS contour on the 3D image was clear. In this study, tomography and volume contrast imaging were used to observe the morphology and integrity of the anal sphincter complex. The delivery group had thinner distal anal sphincter at 12 o’clock, proximal anal sphincter at 6 and 12 o’clock, and central plane at 9 and 12 o’clock versus the C-section group. There was a significant difference between the two groups (P<0.05). There was no significant difference in the thickness of the remaining internal and external sphincter muscle and puborectalis in different directions between the two groups (P> 0.05). The defect of the patient’s vaginal sphincter can be found by three-dimensional ultrasound of the perineum.

## CONCLUSION

PRIM is considered to be another important organizational structure that maintains anal self-control functions in addition to IAS and EAS. Anal sphincter injury is related to PRM injury, and there are many common risk factors for the injury of the two, and the use of forceps to assist labour in normal labour is the most important factor. At present, it is generally accepted that, compared with caesarean section, caesarean delivery has a greater impact on the elevator ani muscles. In this study, there was no statistically significant difference in thickness between the 4 and 8 o’clock planes of the central plane of the caesarean section and the caesarean section. However, there are deficiencies in this study, such as limited sample size, failure to analyse the relationship between ASC thickness and anal incontinence, etc, to be further studied in the future.

### Author`s Contribution:

**NH** conceived the study, literature search, data analysis drafting the manuscript.

**LS** helped in design, data collection, drafting of paper & critical revision ., is also accountable for all aspects of the work in ensuring that questions related to the accuracy or integrity of any part of the work are appropriately investigated and resolved.

***Note:*** Both authors contributed equally.
